# *Enterococcus faecium* bacteraemia: a multicentre observational study focused on risk factors for clinical and microbiological outcomes

**DOI:** 10.1093/jac/dkaf197

**Published:** 2025-06-19

**Authors:** Matteo Rinaldi, Ilaria Rancan, Federica Malerba, Milo Gatti, Leonardo Ancillotti, Beatrice Tazza, Caterina Campoli, Cecilia Bonazzetti, Beatrice Profiti, Natascia Caroccia, Simone Ambretti, Mario Tumbarello, Pierluigi Viale, Maddalena Giannella

**Affiliations:** Department of Medical and Surgical Sciences, Alma Mater Studiorum University of Bologna, Bologna, Italy; Department for Integrated Risk Management, Infectious Disease Unit, IRCCS Azienda Ospedaliero-Universitaria di Bologna, Bologna, Italy; Department of Medical Biotechnologies, University of Siena, Siena, Italy; Department of Medical Sciences, Infectious and Tropical Diseases Unit, Siena University Hospital, Siena, Italy; Department of Medical and Surgical Sciences, Alma Mater Studiorum University of Bologna, Bologna, Italy; Department of Medical and Surgical Sciences, Alma Mater Studiorum University of Bologna, Bologna, Italy; Clinical Pharmacology Unit, IRCCS Azienda Ospedaliero-Universitaria di Bologna, Bologna, Italy; Department of Medical Biotechnologies, University of Siena, Siena, Italy; Department of Medical Sciences, Infectious and Tropical Diseases Unit, Siena University Hospital, Siena, Italy; Department for Integrated Risk Management, Infectious Disease Unit, IRCCS Azienda Ospedaliero-Universitaria di Bologna, Bologna, Italy; Department for Integrated Risk Management, Infectious Disease Unit, IRCCS Azienda Ospedaliero-Universitaria di Bologna, Bologna, Italy; Department of Medical and Surgical Sciences, Alma Mater Studiorum University of Bologna, Bologna, Italy; Department for Integrated Risk Management, Infectious Disease Unit, IRCCS Azienda Ospedaliero-Universitaria di Bologna, Bologna, Italy; Department of Medical and Surgical Sciences, Alma Mater Studiorum University of Bologna, Bologna, Italy; Department for Integrated Risk Management, Infectious Disease Unit, IRCCS Azienda Ospedaliero-Universitaria di Bologna, Bologna, Italy; Department of Medical and Surgical Sciences, Alma Mater Studiorum University of Bologna, Bologna, Italy; Department for Integrated Infectious Risk Management, Operative Unit of Microbiology, IRCCS Azienda Ospedaliero-Universitaria di Bologna, Bologna, Italy; Department of Medical Biotechnologies, University of Siena, Siena, Italy; Department of Medical Sciences, Infectious and Tropical Diseases Unit, Siena University Hospital, Siena, Italy; Department of Medical and Surgical Sciences, Alma Mater Studiorum University of Bologna, Bologna, Italy; Department for Integrated Risk Management, Infectious Disease Unit, IRCCS Azienda Ospedaliero-Universitaria di Bologna, Bologna, Italy; Department of Medical and Surgical Sciences, Alma Mater Studiorum University of Bologna, Bologna, Italy; Department for Integrated Risk Management, Infectious Disease Unit, IRCCS Azienda Ospedaliero-Universitaria di Bologna, Bologna, Italy

## Abstract

**Background:**

Optimal management of *Enterococcus faecium* bloodstream infection (BSI) is not fully understood.

**Methods:**

Multicentre retrospective observational study of all consecutive adult (≥18 years old) patients with *E. faecium* BSI, between January 2016 and December 2023, at two tertiary teaching hospitals in northern Italy. Patients who died within 48 h from BSI onset were excluded. Primary and secondary endpoints were 30 day mortality and persistent *E. faecium* BSI, respectively. Cox regression and logistic binary analyses were used.

**Results:**

Overall, 391 patients were enrolled: median age was 72 (IQR 61–81) years, 225 were male (57.5%), median Charlson comorbidity index (CCI) was 6 (IQR 4–8) and 94 had immunosuppression (24.0%). BSIs were primary, secondary and device-related in 25.1%, 36.2% and 38.7%, respectively. Vancomycin resistance was found in 30.3%. The appropriate empirical therapy rate was given for 29.1%. All-cause 30 day mortality was 34.3% and the rate of persistent BSI was 18.8%. Variables independently associated with 30 day mortality were immunosuppression (HR 1.638, 95% CI 1.022–2.625, *P* = 0.040), SOFA (HR 1.205, 95% CI 1.144–1.268, *P* < 0.001), primary BSI (HR 1.839, 95% CI 1.221–2.770, *P* = 0.004), source control (HR 0.534, 95% CI 0.260–0.972, *P* = 0.042) and the performance of follow-up blood cultures (HR 0.403, 95% CI 0.280–0.972, *P* < 0.001). Factors independently associated with persistent *E. faecium* BSI were: CCI (OR 1.157, 95% CI 1.030–1.300, *P* = 0.014), source control not performed (OR 3.275, 95% CI 1.113–9.635, *P* = 0.031) and teicoplanin (OR 2.023, 95% CI 1.018–4.018, *P* = 0.044).

**Conclusions:**

Among modifiable clinical factors in patients with *E. faecium* BSI, source control and the execution of follow-up blood cultures demonstrated a protective effect on 30 day mortality. Teicoplanin as targeted antibiotic treatment was independently associated with persistent BSI.

## Introduction

Enterococci are emerging causes of community-acquired and healthcare-associated infections.^1^ Despite its minor virulence compared with *Enterococcus faecalis*,^2^  *Enterococcus faecium* has shown an increasing trend as a pathogen in recent years because of its higher levels of antimicrobial resistance, including acquired vancomycin resistance,^1,3,4^ representing a threat and a priority organism for research and drug development.^5^ VRE bloodstream infections (BSIs) are generally associated with high mortality rates regardless of therapeutic management. Indeed, it remains unclear if such increased risk of poor outcome may be mainly related to specific underlying conditions of patients with *E. faecium* bacteriemia compared with patients with other BSIs.^6^ Therefore, it is reasonable to ask if bacteraemia clearance could be a more accurate endpoint in studies involving *E. faecium* BSIs compared with crude mortality. In fact, different studies focused on this topic also considered microbiological endpoints.^7,8^ For these reasons, we tried to evaluate the impact of vancomycin resistance on mortality rates and microbiological clearance.

In addition, there is a relatively high grade of uncertainty about the best therapeutic management of *E. faecium* BSIs. Previous studies identified some risk factors for mortality, such as higher SOFA score or Pitt bacteraemia score,^7,9^ or advantages of high-dose over standard-dose daptomycin;^10^ however, a comprehensive evaluation on management of different types of *E. faecium* BSI (primary, secondary or device-related) is missing. For instance, in the case of *E. faecium* device-related BSI, the real need and duration of antibiotic treatment other than device removal is unclear.^11–13^ As for antibiotic treatment, even if only linezolid has been approved for the treatment of VRE infections, daptomycin is considered one of the key antimicrobial agents for the management of these events, even if its effect seems to be dose-dependent.^14,15^ Daptomycin could have also a role as a backbone agent in combination strategies, especially in isolates with higher MIC values.^15^ Given all these uncertainties about the clinical impact and management of *E. faecium* BSI, we conducted a multicentre retrospective study to shed light on some unclear aspects in the management of *E. faecium* bacteraemia.

## Materials and methods

### Study design, population and setting

This was a multicentre retrospective observational study of all consecutive adult (≥18 years) patients diagnosed with *E. faecium* BSI during hospitalization, over an 8 year period (January 2016 to December 2023), at two tertiary teaching hospitals in northern Italy: IRCCS S. Orsola Hospital (Centre A) and Azienda Ospedaliero-Universitaria Senese, Siena (Centre B). Patients who died within 48 h from BSI onset (see definitions below) were excluded. The maximum follow-up duration was 30 days. The study was approved by the Ethic Committee of the promoting centre (no. 894/2021/Oss/AOUBo). Informed consent was obtained contacting patients via e-mail or phone call. In the case of deceased or unreachable patients, the informed consent was waived considering the observational nature of the study.

### Endpoint variables

Patients were identified through microbiological data and clinical charts. All patients with blood cultures yielding *E. faecium* during the study period were enrolled. The primary endpoint in the overall cohort was 30 day mortality. In addition, in a subgroup of patients with available follow-up blood culture (FUBC), the secondary endpoint was persistence of positive blood cultures. Finally, 30 day mortality was evaluated among patients with device-related BSI, in order to explore variables associated with death.

### Microbiological variables

*E. faecium* BSIs were classified as primary, secondary or device-related according to CDC/NHSN definitions.^16^ Cases in which source of infection was unclear were classified as primary. Persistent *E. faecium* BSI was defined as yielding *E. faecium* from the first retrieved blood cultures within 7 days from the index sample, regardless of appropriate antibiotic treatment. Species identification of positive blood culture bottles was performed from bacterial pellets or subcultured growth by MALDI-TOF MS. Antimicrobial susceptibility was determined using the MicroScan WalkAway semi-automated broth microdilution panels platform, and MIC values were interpreted according to ongoing EUCAST breakpoint tables.

### Main exposure variables

The main exposure variables were both empirical and targeted appropriate antibiotic treatment and source control. Appropriate antibiotic treatment was defined as the administration of at least one drug that was active *in vitro*; daptomycin was considered appropriate in the case of strains with MICs of ≤2 mg/L and if a daily dose of at least ≥10 mg/kg was administered. For the other drugs, vancomycin and linezolid were administered at standard dosing according to renal function; teicoplanin was administered according to a loading dose of 12 mg/kg every 12 h for the first 48 h, then 12 mg/kg daily was maintained with a trough target level between 20 and 30 mg/dL. All these drugs, except daptomycin, were managed with a therapeutic drug monitoring (TDM)-guided approach, resulting in a personalized treatment in each single patient by means of a real-time TDM-guided expert clinical pharmacological advice programme. The first TDM-guided expert clinical pharmacological advice was performed after completing the loading dose period and reassessed every 48–72 h whenever feasible. Teicoplanin *C*_min_ was promptly measured in real time by means of a validated fluorescence polarization immunoassay method, so that TDM-guided expert clinical pharmacological advice for dosing adjustment was provided to clinicians on the same day of blood sampling. An active screening for VRE colonization was not implemented in either of the two centres during the study period. The empirical approach to *E. faecium* BSI was quite similar in both centres and it did not change over the years; generally a glycopeptide was introduced as the first-line agent while awaiting susceptibility testing results. In the case of VRE strains, the choice between daptomycin and linezolid was left to the discretion of the attending physician.

Other exposure variables were demographics, comorbidities according to Charlson comorbidity index (CCI),^17^ classification of BSI and ward of BSI onset. Immunosuppression was defined as the presence of neutropenia, solid organ transplantation, HSCT, chronic steroid treatment (defined as prednisone or equivalent ≥16 mg per day for at least ≥15 days), uncontrolled HIV infection and other forms of immunosuppression (i.e. chemotherapy). Source control was defined as completely performed in the case of optimal management of the source of infection (i.e. device removal or surgical approach in the case of intra-abdominal infection) or partially performed in the case of suboptimal control of source of infection (i.e. percutaneous drainage of intra-abdominal abscess). As for the endpoint, clinical cure was defined as resolution of signs and symptoms, and reduction in SOFA score and biochemical parameters within 7 days from BSI onset and at antibiotic treatment discontinuation.

### Statistical analysis

Categorical variables were reported as counts and percentage. Continuous variables were expressed as mean ± SD if normally distributed, or as median and IQR if non-normally distributed. For the univariate analysis, categorical variables were compared using the χ^2^ test or Fisher’s exact test as appropriate, whereas continuous variables were compared using Student’s *t*-test or the Mann–Whitney *U*-test depending on whether they were normally distributed or not. For the primary endpoint, all-cause mortality, a multivariable Cox regression model was performed. The variables included in the model comprise the main clinical factors considered potentially impactful on 30 day mortality, along with those factors significantly associated with mortality in the univariate analysis. Patients were considered from time 0 (index blood cultures positive for *E. faecium*), to death or 30 days, whichever occurred first. FUBCs and source control were analysed as time-varying covariates.^18^ A binary logistic regression to evaluate risk factors for persistent *E. faecium* BSI was carried out only in patients managed with FUBCs. Finally, a multivariable Cox regression model investigating risk factors for 30 day mortality among patients with device-related BSIs was performed. Statistical significance was considered for *P* values of <0.05. All the analyses were carried out using SPSS 21.00 IBM statistical software.

## Results

Overall, 406 patients were enrolled, after exclusion of 15 patients who died within 48 h from index blood cultures; 391 patients with *E. faecium* BSI were included in the final analysis, of whom 235 (60.1%) were in Centre A and 156 (39.9%) were in Centre B (Table 1). Median age was 72 (IQR 61–81) years, and 225 (57.5%) were male. The median CCI was 6 (IQR 4–8), with approximately one-quarter being immunosuppressed patients (94; 24.0%), and more than half (225; 57.5%) of BSI occurring in medical wards. BSIs were classified as primary, secondary and device-related in 25.3%, 36.1% and 38.6%, respectively. An additional univariate analysis between type of BSI was performed (Table S1, available as Supplementary data at *JAC* Online). The median SOFA and Pitt bacteraemia scores were 4 (IQR 2–6) and 0 (IQR 0–2), respectively. VRE accounted for 30.3% of isolates. BSI was monomicrobial in 252 (65.4%) cases. In 261 (66.8%) patients, FUBCs were collected after a median time of 4 (IQR 2–5) days, yielding the same pathogen in 49/261 (18.8%) cases. As for management of BSI, an attempt at source control was conducted in 176 (45.1%) patients after a median of 3 (IQR 1–6 days). Rates of appropriate empirical and targeted therapy were 29.1% and 97.2%, respectively. Considering targeted treatment, the main drugs administered were linezolid (24.3%), teicoplanin (23.0%), vancomycin (14.8%) and daptomycin (10.2%). All-cause 30 day mortality was 34.3%.

**Table 1.** Comparison between survivors and non-survivors among patients with *E. faecium* BSI

	Survivors *N* = 257 (65.8%)	Non-survivors *N* = 134 (34.2%)	Overall *N* = 391 (100%)	ARR	*P* value
Centre A (Bologna)	163 (63.4)	72 (53.7)	235 (60.1)		0.122
Centre B (Siena)	94 (36.6)	62 (46.3)	156 (39.9)		
Demographic data					
Age (years), median (IQR)	70 (57–78)	75 (67–85)	72 (61–81)		<0.001
Male sex	148 (57.6)	77 (57.5)	225 (57.5)		0.981
Comorbidities					
Myocardial infarction	31 (12.1)	19 (14.2)	50 (12.8)		0.632
Congestive heart failure	30 (11.7)	31 (23.1)	61 (15.6)		0.005
Peripheral vascular disease	19 (7.4)	11 (8.2)	30 (7.7)		0.842
CVA or TIA	32 (12.4)	13 (9.7)	45 (11.5)		0.505
Dementia	28 (10.9)	19 (14.2)	47 (12.0)		0.331
COPD	41 (16.0)	21 (15.7)	62 (15.9)		0.942
Connective tissue disease	12 (4.7)	2 (1.5)	14 (3.6)		0.153
Peptic ulcer disease	19 (7.4)	9 (6.7)	28 (7.1)		0.813
Hemiplegia	6 (2.3)	4 (3.0)	10 (2.6)		0.741
Moderate/severe CKD	53 (20.6)	42 (31.3)	95 (24.3)		0.025
Leukaemia	24 (9.3)	13 (9.7)	37 (9.5)		0.907
Lymphoma	8 (3.1)	7 (5.2)	15 (3.8)		0.405
Liver disease					
Mild	24 (9.3)	6 (4.5)	30 (7.7)		0.109
Moderate/severe	39 (15.2)	10 (7.5)	49 (12.5)		0.036
Diabetes					
Uncomplicated	43 (16.7)	26 (19.4)	69 (17.6)		0.576
End-organ damage	7 (2.7)	1 (0.7)	8 (2.0)		0.273
Solid tumour					
Localized	50 (19.5)	18 (13.4)	68 (17.4)		0.160
Metastatic	26 (10.1)	18 (13.4)	44 (11.2)		0.317
Immunosuppression	64 (24.9)	30 (22.4)	94 (24.0)		0.620
Neutropenia	19 (7.4)	14 (10.4)	33 (8.4)		0.339
SOT	30 (11.7)	7 (5.2)	37 (9.5)		0.045
HSCT	12 (4.7)	2 (1.5)	14 (3.6)		0.152
Chronic steroid treatment	16 (6.2)	13 (9.7)	29 (7.4)		0.227
COVID-19 during hospitalization	45 (17.5)	31 (23.1)	76 (19.4)		0.225
CCI, median (IQR)	5 (4–8)	6 (5–8)	6 (4–8)		0.013
Characteristics of BSI					
Ward of BSI					0.003
Medical	149 (58.0)	76 (56.7)	225 (57.5)		
Surgical	45 (17.5)	8 (6.0)	53 (13.6)		
ICU	38 (14.8)	30 (22.4)	68 (17.4)		
Emergency department	25 (9.7)	20 (14.9)	45 (11.5)		
SOFA score, median (IQR)	3 (1–5)	6 (4–9)	4 (2–6)		<0.001
qSOFA score, median (IQR)	0 (0–1)	1 (0–1)	0 (0–1)		<0.001
Pitt bacteraemia score, median (IQR)	0 (0–1)	2 (0–4)	0 (0–2)		<0.001
Septic shock	16 (6.3)	28 (21.2)	44 (11.4)		<0.001
BSI type					0.001
Primary	51 (19.8)	48 (35.8)	99 (25.3)	16%	
Secondary	105 (40.9)	36 (26.9)	141 (36.1)	−14%	
Intra-abdominal	83 (79.0)	30 (83.3)	113 (80.1)		
UTI	14 (13.3)	5 (13.9)	19 (13.5)		
Other	8 (7.6)	1 (2.8)	9 (6.4)		
Device-related	101 (39.3)	50 (37.3)	151 (38.6)	−2%	
Epidemiological classification					0.051
Community-acquired	11 (4.3)	1 (0.7)	12 (3.1)		
Healthcare-related	37 (14.5)	28 (20.9)	65 (16.6)		
Nosocomial	209 (81.3)	105 (78.4)	314 (80.3)		
Microbiological characteristics					
VRE	75 (29.3)	42 (32.3)	117 (30.3)		0.559
Vancomycin-R, teicoplanin-S	2 (0.8)	1 (0.7)	3 (0.8)		0.846
Vancomycin-S, Teicoplanin-R	4 (1.6)	3 (2.3)	7 (1.8)		0.546
Vancomycin-R, teicoplanin-R	73 (28.4)	41 (30.6)	114 (29.2)		0.762
Daptomycin MIC ≤ 2 mg/L	109 (64.5)	46 (67.6)	155 (65.4)		0.763
Daptomycin MIC > 2 mg/L	60 (35.5)	22 (32.4)	82 (34.6)		
Linezolid MIC < 2 mg/L	14 (5.4)	9 (6.7)	25 (6.4)		0.228
Linezolid MIC = 2 mg/L	86 (50.9)	34 (50.0)	120 (50.6)		
Linezolid MIC > 2 mg/L	69 (26.8)	25 (36.8)	94 (24.0)		
Monomicrobial	172 (66.9)	81 (62.3)	252 (65.4)		0.368
Polymicrobial	85 (33.1)	49 (37.7)	134 (34.6)		0.259
Enterobacteriaceae	41 (48.8)	17 (34.7)	58 (43.6)		
Non-fermenting Gram negative	5 (6.0)	7 (14.3)	12 (9.0)		
CoNS	25 (29.8)	14 (28.6)	39 (29.3)		
*S. aureus*	1 (1.2)	2 (4.1)	3 (2.3)		
*Candida* spp.	12 (14.3)	9 (18.4)	21 (15.8)		
Management of BSI					
FUBC	195 (75.9)	66 (49.3)	261 (66.8)	−26.6%	<0.001
*E. faecium*, same profile	28 (14.4)	12 (18.2)	40 (15.3)		0.191
*E. faecium*, different profile	6 (3.1)	3 (4.5)	9 (3.4)		
Another pathogen	22 (11.3)	13 (19.7)	35 (13.4)		
Negative	139 (71.3)	38 (57.6)	177 (67.8)		
Time from BSI to FUBC (days), median (IQR)	3 (2–5)	4 (2–6)	4 (2–5)		0.155
Time from BSI to negative FUBC (days), median (IQR)	4 (3–6)	5 (3–7)	5 (3–6)		0.414
Echocardiographic study	160 (62.7)	37 (28.0)	197 (50.9)		<0.001
Positive	8 (5.0)	1 (2.7)	9 (4.6)		0.546
Ultrasound study	42 (16.4)	10 (7.6)	52 (13.4)		0.041
Positive	15 (35.7)	4 (40.0)	19 (36.5)		0.800
Source control					<0.001
Completely performed	124 (48.2)	34 (25.6)	158 (40.5)	−22.6%	
Partially performed	17 (6.6)	1 (0.8)	18 (4.6)		
Not performed	30 (11.7)	39 (29.3)	69 (17.7)	17.6%	
Not applicable	86 (33.5)	59 (44.4)	145 (37.2)		
Time from BSI to source control (days), median (IQR)	3 (1–7)	3 (1–4)	3 (1–6)		0.475
Antibiotic treatment					
Empirical treatment^a^	214 (83.3)	121 (90.3)	335 (85.7)		0.060
Appropriate empirical therapy^b^	57 (26.6)	40 (33.6)	97 (29.1)		0.208
Vancomycin	14 (58.3)	19 (82.6)	33 (70.2)		0.069
Teicoplanin	10 (41.7)	4 (17.4)	14 (29.8)		0.111
Daptomycin	12 (21.1)	8 (20.0)	20 (20.6)		0.900
Linezolid	21 (36.8)	9 (22.5)	30 (30.9)		0.181
Duration of empirical therapy (days), median (IQR)	2 (1–2)	2 (1–3)	2 (1–2)		0.245
Targeted treatment	193 (75.7)	94 (70.7)	287 (74.0)		0.330
Vancomycin	37 (14.4)	21 (15.7)	58 (14.8)		0.765
CI after LD	12 (32.4)	4 (20.0)	16 (28.1)		0.206
Intermittent infusion	22 (59.5)	16 (80.0)	38 (66.7)		
Extended infusion	3 (8.1)	0 (0)	3 (5.3)		
Teicoplanin	70 (27.2)	20 (14.9)	90 (23.0)		0.006
Daptomycin	23 (8.9)	17 (12.7)	40 (10.2)		0.292
<10 mg/kg	8 (36.4)	12 (75.0)	20 (52.6)		0.019
≥10 mg/kg	14 (63.6)	4 (25.0)	18 (47.4)		
Linezolid	61 (23.7)	34 (25.4)	95 (24.3)		0.711
Monotherapy	143 (55.6)	72 (53.7)	215 (55.0)		0.749
Combination therapy	50 (19.5)	22 (16.4)	72 (18.4)		
β-Lactam	44 (17.1)	20 (14.9)	64 (16.4)		0.666
Fosfomycin	3 (1.2)	1 (0.7)	4 (1.0)		0.695
Appropriate targeted therapy^b^	187 (97.4)	88 (96.7)	275 (97.2)		0.743
Duration of appropriate therapy (days), median (IQR)	11 (7–16)	7 (3–11)	10 (5–15)		<0.001
Adverse event	13 (7.3)	3 (3.7)	16 (6.2)		0.404
Outcome					
Clinical cure (by Day 7)	210 (81.7)	14 (10.4)	224 (57.3)		<0.001
SOFA score (by Day 7)	2 (1–4)	5 (0–8)	2 (0–5)		<0.001
Breakthrough BSI^c^	8 (3.1)	1 (0.7)	9 (2.3)		0.174
Recurrent BSI^d^	58 (22.6)	7 (5.2)	65 (16.6)		<0.001
LOS (days), median (IQR)	41 (22–65)	23 (13–38)	34 (18–58)		<0.001
Time from BSI onset to death (days), median (IQR)			9 (4–16)		

Values are *n* (%) unless otherwise stated. ARR, absolute risk reduction; CVA, cerebrovascular accident; TIA, transient ischaemic attack; CKD, chronic kidney disease; qSOFA, quick SOFA; R, resistant; S, susceptible; UTI, urinary tract infection; CI, continuous infusion; LD, loading dose; LOS, length of stay.

^a^Defined as any antimicrobial therapy.

^b^Appropriate therapy was defined as the administration of at least one drug that was *in vitro* active.

^c^Breakthrough BSI was defined as a new-onset BSI occurring after a negative FUBC during appropriate antibiotic treatment.

^d^Recurrent BSI was defined as a new BSI episode occurring in a period of more than 72 h after antibiotic withdrawal.

### Risk factors for 30 day mortality among the overall population

**Figure 1.**
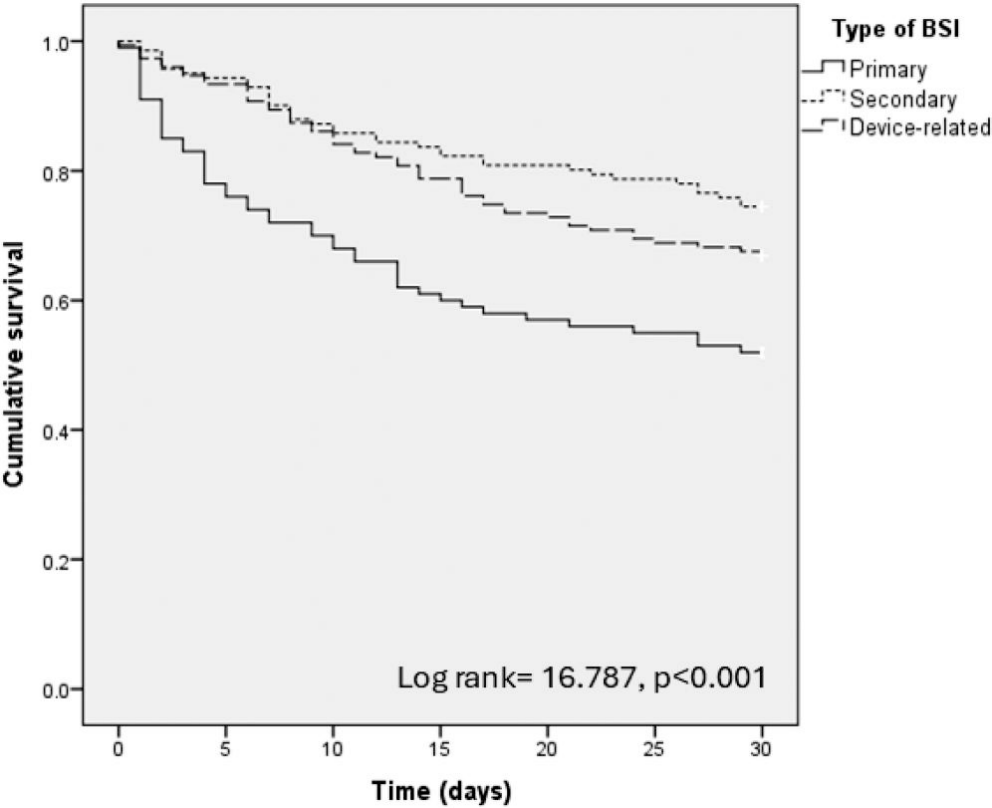
Kaplan–Meier curves for 30 day mortality among different types of BSI.

A comparison between 30 day survivors and non-survivors was performed (Table 1). Significant differences were observed for age (70 versus 75 years, *P* < 0.001), CCI (5 versus 6, *P* = 0.013) and solid organ transplant (SOT) (11.7% versus 5.2%, *P* = 0.045), BSI onset in ICU (14.8% versus 22.4%, *P* = 0.003), higher SOFA score (3 versus 6, *P* < 0.001) and Pitt bacteraemia score (0 versus 2, *P* < 0.001). Primary BSIs had the highest rates of death (19.8% versus 35.3%), whereas secondary BSI had the lowest (40.9% versus 27.1%, *P* = 0.001) (Table 1 and Figure 1). No differences among resistances profiles or between monomicrobial and polymicrobial BSI were observed. Mortality was higher among patients in whom source control was not performed (29.3% versus 11.7%, *P* < 0.001) (Figure S1). No differences between appropriate empirical and targeted therapy, or monotherapy and combination therapy were observed. At multivariable analysis adjusted for main risk factors (see Table 2), immunosuppression (HR 1.638, 95% CI 1.022–2.625, *P* = 0.040), SOFA score (HR 1.205, 95% CI 1.144–1.268, *P* < 0.001) and primary BSI (HR 1.839, 95% CI 1.221–2.770, *P* = 0.004) were independently associated with 30 day mortality. Performing adequate source control (HR 0.534, 95% CI 0.260–0.972, *P* = 0.042) and execution of FUBC (HR 0.403, 95% CI 0.280–0.972, *P* < 0.001) were protective (Figure S2).

**Table 2.** Cox regression for 30 day mortality adjusted for sex, CCI, immunosuppression, ward of BSI onset, SOFA score, type of BSI, VRE phenotype, mono/polymicrobial BSI, appropriate empirical therapy, appropriate targeted therapy, execution of FUBC and source control

Variable	HR	95% CI	*P* value
Male gender	0.761	(0.534–1.084)	0.130
CCI	1.059	(0.990–1.133)	0.097
Immunosuppression	1.638	(1.022–2.625)	0.040
BSI onset in ICU	0.883	(0.514–1.514)	0.650
Monomicrobial BSI	1.032	(0.708–1.505)	0.871
VRE	1.187	(0.820–1.719)	0.364
Primary BSI	1.839	(1.221–2.770)	0.004
SOFA score	1.205	(1.144–1.268)	<0.001
Appropriate empirical therapy	0.859	(0.575–1.284)	0.458
Appropriate targeted therapy	0.841	(0.574–1.230)	0.372
FUBC^a^	0.403	(0.280–0.581)	<0.001
Source control^a^	0.534	(0.260–0.972)	0.042

^a^Expressed as a time-varying variable.

### Risk factors for persistent E. faecium BSI

Overall, FUBCs were performed in 261 patients, with a median time of 5 (IQR 2–5) days from index BSI. In 49 (18.8%) cases, a persistent *E. faecium* BSI was demonstrated. A comparative analysis of patients with and without persistent BSI was performed (Table S2). Time from index blood cultures to negative FUBC was 6 (IQR 4.75–6) and 4 (IQR 3–6) days (*P* < 0.001) in patients with and without persistent BSI. Patients with persistent *E. faecium* BSI showed a higher median CCI (7 versus 5, *P* = 0.011). Secondary BSIs were associated with persistent bacteraemia (49.0% versus 34.4%, *P* = 0.051); however, no differences regarding pattern of susceptibility, monomicrobial/polymicrobial bacteraemia and source control were reported between groups. As for antibiotic treatment, only teicoplanin as targeted treatment was associated with persistent BSI (44.9% versus 29.2%, *P* = 0.042) (Figure S3); clinical cure rates at Day 7 were lower (55.1% versus 70.3%, *P* = 0.044) and length of hospital stay higher (50 versus 39 days, *P* = 0.028). No differences in 30 day mortality and time from BSI onset to death were observed. At logistic regression, adjusted for main covariates (Table 3), CCI (OR 1.157, 95% CI 1.030–1.300, *P* = 0.014), source control not being performed (OR 3.275, 95% CI 1.113–9.635, *P* = 0.031) and teicoplanin as target treatment (OR 2.023, 95% CI 1.018–4.018, *P* = 0.044) were associated with persistent *E. faecium* BSI.

**Table 3.** Binary logistic regression for persistent *E. faecium* BSI adjusted for CCI, type of BSI, appropriate empirical therapy, targeted therapy and source control

Variable	OR	95% CI	*P* value
CCI	1.157	(1.030–1.300)	0.014
Source control not performed	3.275	(1.113–9.635)	0.031
Secondary BSI	2.042	(0.749–5.568)	0.163
Appropriate empirical therapy	1.216	(0.536–2.757)	0.219
Teicoplanin as targeted therapy	2.023	(1.018–4.018)	0.044

### Risk factors for 30 day mortality among patients with device-related E. faecium BSI

**Figure 2.**
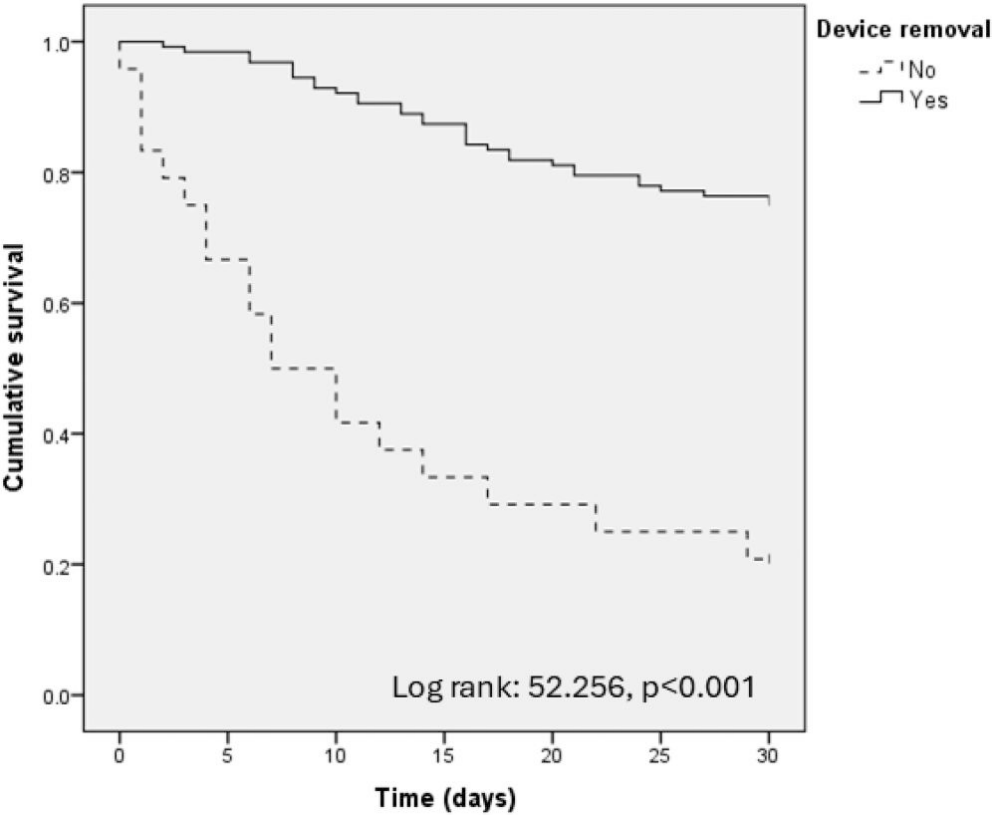
Kaplan–Meier curves for 30 day mortality among patients with device-related BSI.

Overall, 151 patients had a device-related *E. faecium* BSI, of whom 50 (33.1%) died within 30 days after index BCs (Table S3). Differences between survivors and non-survivors included age (68 versus 72 years, *P* = 0.018) and neutropenia (5.9% versus 20.0%, *P* = 0.012), BSI onset in ICU (20.8% versus 36.0%, *P* = 0.053) and higher SOFA score (3 versus 6, *P* < 0.001). Performance of echocardiographic study (60% versus 32%, *P* = 0.002), vein/arterial ultrasound (31.7% versus 16.3%, *P* = 0.085) and device removal (95% versus 62%, *P* < 0.001) were associated with lower mortality rates. No difference in rates of both overall empirical and targeted therapy were observed; however, targeted treatment with daptomycin was associated with higher 30 day mortality (5.0% versus 20.0%, *P* = 0.007). Source control was strongly associated with improved survival (Figure 2). At multivariable analysis adjusted for main covariates, SOFA score (HR 1.165, 95% CI 1.089–1.217, *P* < 0.001) was associated with death, whereas execution of FUBC (HR 0.435, 95% CI 0.217–0.873, *P* = 0.019) and device removal (HR 0.229, 95% CI 0.117–0.451, *P* < 0.001) were protective (Table 4).

**Table 4.** Cox regression for 30 day mortality among patients with device-related *E. faecium* BSI adjusted for CCI, SOFA score, FUBC, appropriate empirical therapy, targeted therapy and source control

Variable	OR	95% CI	*P* value
CCI	1.110	(0.994–1.240)	0.063
SOFA score	1.165	(1.089–1.247)	<0.001
Appropriate empirical therapy	0.916	(0.501–1.673)	0.775
Appropriate targeted therapy	1.213	(0.609–2.417)	0.584
FUBC^a^	0.435	(0.217–0.873)	0.019
Device removal^a^	0.229	(0.117–0.451)	<0.001

^a^Expressed as a time-varying variable.

## Discussion

Our study highlights some aspects of the management of *E. faecium* BSI not previously clarified. Firstly, primary BSIs are associated with the highest risk of mortality, since the mortality risk in both secondary and device-related BSIs could be mitigated by appropriate source control. Immunosuppressed state may increase such risk. Persistent *E. faecium* BSI is driven by lack of source control and targeted teicoplanin treatment. Although in our study persistent BSI was not predictive of mortality, it could be indicative of suboptimal patient management. For device-related BSIs, device removal is the cornerstone of optimal management, whereas appropriate antibiotic treatment may have a minor impact on mortality.

*E. faecium* BSIs have increasingly become frequent causes of healthcare-associated infections, representing the third most common pathogen among Gram-positive BSIs.^19^ Although it is unclear whether unfavourable outcomes are directly caused by *E. faecium* BSI or whether it is a marker of underlying poor underlying conditions, mortality rates remain high. In our cohort, the overall 30 day mortality was 34.3%, similar to previous cohorts.^20,21^ As reported by the WHO, VRE is rapidly becoming a global challenge in the context of increasing vancomycin resistance observed worldwide.^5^ The lack of a first-line treatment in VRE infections could be one of the drivers of poor outcomes in such population. However, our results seem to suggest that vancomycin resistance itself is not associated with poor outcome, as 30 day mortality among patients with vancomycin-susceptible *Enterococcus* and VRE BSI were 33.6% and 35.6%, respectively. As older patients and those with immunosuppression are at higher risk of death, both empirical and targeted appropriate therapy were not associated with increased survival rates. In the overall cohort, patients in whom source control was not performed were at highest risk of death, highlighting the need for prompt identification of infection source. Accordingly, patients in whom infection source was not identifiable or in whom source control was not applicable (i.e. primary BSIs) were at high risk of death. After adjustment for confounders, we found that the execution of FUBC could have a protective effect on mortality, both in the overall cohort and in the subgroup of patients with device-related BSI. Since the performance of FUBC was not a standardized approach and may be influenced by individual patient characteristics, this finding must be interpreted with caution and should be the subject of future research. Several studies demonstrated that persistent bacteraemia, especially for *Staphylococcus aureus* and Gram-negative bacteria, is associated with increased risk of death. Accordingly, a multicentre study found that persistent enterococcal bacteraemia is an independent risk factor for 30 day mortality.^22^ Authors identified patients with haematological malignancies and initial treatment with daptomycin as independent risk factors for persistent bacteraemia. However, more than half of isolates were *E. faecalis* susceptible to a β-lactam agent, and dose of daptomycin administered was not available. In our study, we found some modifiable factors associated with persistent *E. faecium* BSIs, such as performing appropriate source control and targeted treatment with teicoplanin. Regarding the latter issue, only a few studies found that teicoplanin is a safe alternative to vancomycin for glycopeptide-susceptible *E. faecium* BSI, resulting in similar rates of 30 day mortality.^23–25^ However, no previous studies explored the impact of appropriate targeted treatment on risk of persistent BSI. It is worth noting that in our study, treatment with teicoplanin was found to be associated with persistent BSI despite no significant differences in trough concentrations after the loading dose between the two groups (23% versus 23%, *P* = 0.996). Considering the peculiar pharmacokinetic features (a very high plasma protein binding and a steady state reached after 4–5 administrations),^26^ teicoplanin may result in a prolonged time of bacteraemia clearance. However, teicoplanin use was not associated with an increased risk of death.

Management of *E. faecium* device-related BSI is historically an unmet need. Guidelines recommend device removal, along with targeted antibiotic therapy, without any distinction between *E. faecalis* and *E. faecium.*^27^ However, *E. faecium* is generally considered as a microorganism with a low level of pathogenicity, unfrequently causing complicated BSIs. Indeed, it is estimated that only 1% of patients with *E. faecium* BSI may develop endocarditis, compared with more than 10% of those with *E. faecalis* BSIs.^28^ Thus, probably microbiological differences between *E. faecalis* and *E. faecium* can justify a different approach when managing catheter-related BSI (CR-BSI). In *E. faecium* CR-BSI, source control alone could be the only therapeutic measure, as our study demonstrated a significant impact on patient survival. Few indirect data supporting such management are available, even in special populations such as paediatric stem cell transplant patients or patients on haemodialysis.^29,30^ Among cancer patients with mixed enterococcal CR-BSI (50% *E. faecium*), early (<3 days) catheter removal was associated with a trend toward better overall outcome.^31^ Nevertheless, further studies addressing this specific issue are urgently required, potentially identifying a subgroup of patients in whom antibiotic exposure could be unnecessary.

The present research has intrinsic limitations due to the observational retrospective design. However, data quality checking was performed in both centres. The administered drugs were established by attending physicians, thus selection bias should be recognized. To address this issue, we adjusted multivariable models for the most relevant and significant covariates that could have influenced both the therapeutic management and the clinical outcome, but some unrecognized factors could have occurred. We preferred to exclude patients who died within 48 h from the index blood culture because targeted antibiotic treatment is usually delayed, as species identification and susceptibility testing may require up to 1–2 days to be available. Considering that in less than 30% of included patients an appropriate empirical treatment was introduced, including patients who died within 48 h could underestimate the benefit of appropriate targeted therapy. Moreover, active surveillance for VRE colonization was not implemented in either of the two centres, which could result in further delays in administration of effective empirical and targeted therapy. Again, performance of FUBC and source control, including catheter removal, were done at the discretion of attending physicians, with both selection and immortal bias maybe occurring. To address the immortal bias, both such variables were used as time-varying in the multivariable models.

To conclude, in our large cohort of patients with *E. faecium* BSIs, primary BSI and immunosuppression are independent predictors of death. Source control and execution of FUBC could mitigate such a risk. Among patients with device-related *E. faecium* BSI, device removal is the cornerstone of optimal management. Considering that both appropriate empirical and targeted treatment seem not to impact mortality in patients with device-related BSI, future studies identifying subgroups of patients in whom antibiotic treatment could be unnecessary are strongly advocated.

## Supplementary Material

dkaf197_Supplementary_Data

## Supplementary data

Figures S1 to S3 and Tables S1 to S3 are available as Supplementary data at *JAC* Online.
