# Supplementary material for: Enterococcus faecium bacteraemia: a multicentre observational study focused on risk factors for clinical and microbiological outcomes
Source: J Antimicrob Chemother. 2025 Jun 19;80(8):2247–56. doi: 10.1093/jac/dkaf197 (PMC12313462; doi:10.1093/jac/dkaf197)
Supplement: dkaf197_Supplementary_Data [file dkaf197_supplementary_data.zip › Suppl. Tables_final.docx]

**Table S1**. Univariate analysis between type of *E. faecium* BSI.

|  | **Primary**  **N=99 (25.3%)** | **Secondary**  **N=141 (36.1%)** | **Device-related**  **N=151 (38.6%)** | **p-value** |
| --- | --- | --- | --- | --- |
| **Demographic data** |  |  |  |  |
| Age (years) (median, IQR) | 76 (64 -85) | 72 (64-82) | 69 (56-78) | 0.002 |
| Male sex | 60 (60.6) | 74 (52.5) | 92 (60.9) | 0.289 |
| **Comorbidities** |  |  |  |  |
| Immunosuppression | 26 (26.3) | 27 (19.1) | 41 (27.2) | 0.228 |
| CCI (median, IQR) | 6 (4-8) | 6 (5-8) | 5 (3-8) | 0.005 |
| **Characteristics of BSI** |  |  |  |  |
| Ward of BSI |  |  |  |  |
| Medical | 62 (62.6) | 79 (56.0) | 84 (55.6) | <0.001 |
| Surgical | 9 (9.1) | 24 (17.1) | 20 (13.2) |  |
| ICU | 13 (13.1) | 16 (11.3) | 39 (25.8) |  |
| Emergency department | 15 (15.2) | 22 (15.6) | 8 (5.3) |  |
| SOFA score (median, IQR) | 4 (3-8) | 3 (2-5) | 4 (2-6) | 0.006 |
| qSOFA score (median, IQR) | 0 (0-1) | 0 (0-1) | 1 (0-2) | 0.007 |
| PITT score (median, IQR) | 1 (0-2) | 0 (0-1) | 1 (0- 2) | 0.007 |
| Septic shock | 14 (14.4) | 16 (11.4) | 14 (9.4) | 0.480 |
| Epidemiological classification |  |  |  |  |
| Nosocomial | 73 (73.7) | 106 (75.7) | 133 (88.7) | 0.001 |
| **Microbiological characteristics** |  |  |  |  |
| VRE | 33 (33.3) | 41 (29.1) | 44 (29.1) | 0.736 |
| Monomicrobial | 65 (65.7) | 97 (68.8) | 95 (62.9) | 0.569 |
| Polymicrobial | 34 (34.3) | 44 (31.2) | 56 (37.1) |  |
| **Management of BSI** |  |  |  |  |
| FUBC | 61 (61.6) | 97 (68.8) | 102 (67.5) | 0.489 |
| Time from BSI to FUBC | 3 (2-5) | 5 (5-6) | 4 (2-5) | 0.577 |
| Time from BSI to negative FUBC | 4 (3-6) | 5 (3-6) | 4 (3-6) | 0.676 |
| Source control |  |  |  |  |
| Performed | 0 (0) | 36 (32.7) | 127 (84.1) | <0.001 |
| Not performed | 0 (0) | 42 (29.8) | 24 (15.9) |  |
| Time from BSI to source control (days) (median, IQR) | - | 4 (1-7) | 3 (1-5) | 0.057 |
| **Antibiotic treatment** |  |  |  |  |
| Appropriate empirical therapy | 29 (34.9) | 23 (17.6) | 46 (38.7) | <0.001 |
| Targeted treatment | 65 (65.7) | 111 (79.9) | 111 (74.0) | 0.050 |
| Vancomycin | 15 (15.2) | 24 (17.0) | 19 (12.6) | 0.796 |
| Teicoplanin | 15 (15.2) | 40 (28.4) | 34 (22.5) | 0.054 |
| Daptomycin | 10 (10.1) | 15 (10.6) | 15 (9.9) | 0.977 |
| Linezolid | 24 (24.2) | 31 (22.0) | 41 (27.2) | 0.600 |
| Monotherapy | 54 (54.5) | 75 (53.2) | 86 (57.0) | 0.804 |
| Combination therapy | 11 (11.1) | 36 (25.5) | 25 (16.6) | 0.487 |
| Appropriate targeted therapy | 60 (95.2) | 107 (98.2) | 108 (97.3) | 0.585 |
| Duration of appropriate therapy (days) (median, IQR) | 9 (1- 14) | 11 (6-16) | 10 (6-14) | 0.063 |
| **Outcome** |  |  |  |  |
| Clinical cure (+7-day) | 51 (51.5) | 77 (54.6) | 96 (63.6) | 0.120 |
| SOFA score (+7-day) | 2 (0-5) | 2 (0-4) | 3 (1-6) | <0.001 |
| Persistent BSI | 12 (19.7) | 24 (24.7) | 12 (11.8) | 0.168 |
| Breakthrough BSI | 0 (0) | 5 (3.5) | 4 (2.6) | 0.165 |
| Recurrent BSI | 12 (12.1) | 23 (16.3) | 30 (19.9) | 0.279 |
| LOS (days) (median, IQR) | 24 (15-46) | 29 (17-56) | 40 (25-79) | <0.001 |
| All-cause 30-day mortality | 48 (48.5) | 36 (25.5) | 50 (33.1) | 0.001 |
| Time from BSI onset to death (days) (median, IQR) | 6 (2-13) | 10 (6-20) | 11 (6-17) | 0.018 |

**Abbreviations:** CCI Charlson comorbidity score, BSI bloodstream infection, ICU intensive care unit, FUBC follow-up blood cultures, LOS length of hospital stay.

**Table S2**. Univariate analysis among patients which performed FUBC with persistent vs. negative FUBC.

|  | **Persistent BSI**  **N=49 (18.8%)** | **Negative FUBC**  **N=212 (81.2%)** | **Overall**  **N=261 (100%)** | **p-value** |
| --- | --- | --- | --- | --- |
| Center A (Bologna) | 40 (81.6) | 151 (71.2) | 191 (73.2) | 0.156 |
| Center B (Siena) | 9 (18.4) | 61 (28.8) | 70 (26.8) |  |
| **Demographic data** |  |  |  |  |
| Age (years) (median, IQR) | 73 (63-81) | 69 (57-78) | 70 (59-78) | 0.079 |
| Male sex | 33 (67.3) | 115 (54.2) | 148 (56.7) | 0.111 |
| **Comorbidities** |  |  |  |  |
| Myocardial infarction | 7 (14.3) | 24 (11.3) | 31 (11.9) | 0.624 |
| Congestive heart failure | 10 (20.4) | 25 (11.8) | 35 (13.5) | 0.160 |
| Peripheral vascular disease | 9 (18.4) | 18 (8.5) | 27 (10.3) | 0.064 |
| CVA or TIA | 8 (16.3) | 25 (11.8) | 33 (12.6) | 0.473 |
| Dementia | 7 (14.3) | 20 (9.4) | 27 (10.3) | 0.305 |
| COPD | 7 (14.3) | 33 (15.6) | 40 (15.3) | 0.511 |
| Connective tissue disease | 2 (4.1) | 8 (3.8) | 10 (3.8) | 1.000 |
| Peptic ulcer disease | 4 (8.2) | 16 (7.5) | 20 (7.7) | 0.774 |
| Hemiplegia | 3 (6.1) | 5 (2.4) | 8 (3.1) | 0.174 |
| Moderate/severe CKD | 13 (26.5) | 43 (20.3) | 56 (21.5) | 0.339 |
| Leukemia | 8 (16.3) | 21 (9.9) | 29 (11.1) | 0.210 |
| Lymphoma | 1 (2.0) | 10 (4.7) | 11 (4.2) | 0.695 |
| Liver disease |  |  |  |  |
| Mild | 7 (14.3) | 18 (8.5) | 25 (9.6) | 0.278 |
| Moderate/severe | 8 (16.3) | 29 (13.7) | 37 (14.2) | 0.389 |
| Diabetes |  |  |  |  |
| Uncomplicated | 11 (22.4) | 35 (16.5) | 46 (17.6) | 0.405 |
| End-organ damage | 1 (2.0) | 6 (2.8) | 7 (2.7) | 1.000 |
| Solid tumor |  |  |  |  |
| Localized | 10 (20.4) | 36 (17.0) | 46 (17.6) | 0.539 |
| Metastatic | 6 (12.2) | 23 (10.8) | 29 (11.1) | 0.802 |
| Immunosuppression | 18 (36.7) | 54 (25.5) | 72 (27.6) | 0.115 |
| Neutropenia | 7 (14.3) | 21 (9.9) | 28 (10.7) | 0.441 |
| SOT | 9 (18.4) | 20 (9.4) | 29 (11.1) | 0.081 |
| HSCT | 1 (2.0) | 12 (5.7) | 13 (5.0) | 0.473 |
| Chronic steroid treatment | 3 (6.1) | 13 (6.1) | 16 (6.1) | 1.000 |
| COVID-19 during hospitalization | 11 (22.4) | 41 (19.3) | 52 (19.9) | 0.692 |
| CCI (median, IQR) | 7 (5-9) | 5 (4-8) | 5 (4-8) | 0.011 |
| **Characteristics of BSI** |  |  |  |  |
| Ward of BSI |  |  |  | 0.169 |
| Medical | 32 (65.3) | 123 (58.0) | 155 (59.4) |  |
| Surgical | 9 (18.4) | 27 (12.7) | 36 (13.8) |  |
| ICU | 7 (14.3) | 40 (18.9) | 47 (18.0) |  |
| Emergency department | 1 (2.0) | 22 (10.4) | 23 (8.8) |  |
| SOFA score (median, IQR) | 4 (2-5-5) | 3 (2-6) | 4 (2-6) | 0.911 |
| qSOFA score (median, IQR) | 0 (0-1) | 0 (0-1) | 0 (0-1) | 0.709 |
| PITT score (median, IQR) | 0 (0-1) | 0 (0-2) | 0 (0-1) | 0.093 |
| Septic shock | 2 (4.2) | 20 (9.5) | 22 (8.5) | 0.388 |
| BSI type |  |  |  | 0.051 |
| Primary | 13 (26.5) | 49 (23.1) | 62 (23.8) |  |
| Secondary | 24 (49.0) | 73 (34.4) | 97 (37.2) | 0.603 |
| Intra-abdominal | 21 (87.5) | 58 (79.5) | 79 (81.4) |  |
| UTI | 1 (4.2) | 9 (12.3) | 10 (10.3) |  |
| Other | 2 (8.3) | 6 (8.2) | 8 (8.2) |  |
| Device-related | 12 (24.5) | 90 (42.5) | 102 (39.1) |  |
| Epidemiological classification |  |  |  | 0.045 |
| Community-acquired | 0 (0.0) | 9 (4.3) | 9 (3.5) |  |
| Healthcare-related | 3 (6.1) | 36 (17.1) | 39 (15.0) |  |
| Nosocomial | 46 (93.9) | 166 (78.7) | 212 (81.5) |  |
| **Microbiological characteristics** |  |  |  |  |
| VRE | 15 (30.6) | 65 (30.7) | 80 (30.7) | 1.000 |
| Monomicrobial | 34 (69.4) | 138 (65.1) | 172 (65.9) | 0.619 |
| Polymicrobial | 15 (30.6) | 74 (34.9) | 89 (34.1) |  |
| **Management of BSI** |  |  |  |  |
| Time from BSI to FUBC | 3 (2-5) | 4 (2-5) | 5 (2-5) | 0.062 |
| Time from BSI to negative FUBC | 6 (4.75-6) | 4 (3-6) | 5 (3-6) | <0.001 |
| Echocardiographic study | 31 (63.3) | 136 (64.5) | 167 (64.2) | 0.870 |
| Positive | 2 (6.5) | 6 (4.4) | 8 (4.8) | 0.457 |
| Ultrasound study | 9 (18.4) | 39 (18.5) | 48 (18.5) | 0.977 |
| Positive | 4 (44.4) | 12 (30.8) | 16 (33.3) | 0.457 |
| Source control |  |  |  | 0.146 |
| Performed | 21 (42.9) | 110 (51.9) | 131 (50.2) |  |
| Not performed | 11 (22.4) | 25 (11.8) | 36 (13.8) |  |
| Not applicable | 17 (34.7) | 77 (36.3) | 94 (36.0) |  |
| Time from BSI to source control (days) (median, IQR) | 4 (2.5-9) | 3 (1-5) | 3 (1-6) | 0.009 |
| **Antibiotic treatment** |  |  |  |  |
| Empirical treatment | 42 (85.7) | 175 (82.5) | 217 (83.1) | 0.677 |
| Appropriate empirical therapy | 10 (24.4) | 47 (26.9) | 57 (26.4) | 0.845 |
| Targeted treatment | 45 (91.8) | 174 (82.9) | 219 (84.6) | 0.130 |
| Vancomycin | 6 (12.2) | 24 (11.3) | 30 (11.5) | 0.807 |
| CI after LD | 6 (100) | 8 (33.3) | 14 (46.7) | 0.022 |
| Intermittent infusion | 0 (0) | 13 (54.2) | 13 (43.3) |  |
| Extended infusion | 0 (0) | 3 (12.5) | 3 (10.0) |  |
| Teicoplanin | 22 (44.9) | 62 (29.2) | 84 (32.2) | 0.042 |
| Underexposure after LD | 19 (27.1) | 4 (20.0) | 23 (25.6) | 0.518 |
| Teicoplanin trough value (mg/dL) (median, IQR) | 23 (19-27) | 23 (20-24) | 23 (19-27) | 0.996 |
| Daptomycin | 7 (16.3) | 23 (11.3) | 30 (12.3) | 0.338 |
| <10 mg/Kg | 4 (57.1) | 13 (56.5) | 17 (56.7) | 0.977 |
| ≥10 mg/Kg | 3 (42.9) | 10 (43.5) | 13 (43.3) |  |
| Linezolid | 9 (18.4) | 61 (28.8) | 70 (26.8) | 0.156 |
| Monotherapy | 36 (73.5) | 135 (63.7) | 171 (65.5) | 0.243 |
| Combination therapy | 9 (18.4) | 39 (18.4) | 48 (18.4) | 1.000 |
| Beta-lactam | 7 (14.3) | 34 (16.0) | 41 (15.7) | 1.000 |
| Fosfomycin | 3 (6.1) | 0 (0.0) | 3 (1.1) | 0.006 |
| Appropriate targeted therapy | 45 (100.0) | 165 (95.9) | 210 (96.8) | 0.349 |
| Duration of appropriate therapy (days) (median, IQR) | 14 (9.25-20) | 10 (6.5-15) | 11 (7-15) | 0.004 |
| Adverse event | 3 (6.8) | 8 (5.1) | 11 (5.5) | 0.709 |
| **Outcome** |  |  |  |  |
| Clinical cure (+7-day) | 27 (55.1) | 149 (70.3) | 176 (67.4) | 0.044 |
| SOFA score (+7-day) | 4 (1-5) | 3 (1-5) | 3 (1-5) | 0.186 |
| Breakthrough BSI | 3 (6.1) | 4 (1.9) | 7 (2.7) | 0.125 |
| Recurrent BSI | 10 (20.4) | 45 (21.2) | 55 (21.1) | 1.000 |
| 30-day mortality | 15 (30.6) | 51 (24.1) | 66 (25.3) | 0.364 |
| 90-day mortality | 26 (53.1) | 82 (38.7) | 108 (41.4) | 0.077 |
| LOS (days) (median, IQR) | 50 (34.5-76.5) | 39 (23-64) | 42 (23-65) | 0.028 |
| Time from BSI to death (days) (median, IQR) | 15 (10-22) | 14 (10-22) | 14.5 (10-22) | 0.896 |

**Abbreviations:** CVA cerebrovascular accident, TIA transient ischemic attack, COPD chronic obstructive pulmonary disease, CKD chronic kidney disease, SOT solid organ transplant, HSCT hematologic stem cell transplant, CCI Charlson comorbidity score, BSI bloodstream infection, ICU intensive care unit, UTI urinary tract infection, FUBC follow-up blood cultures, CI continuous infusion, LD loading dose.

**Table S3**. Comparison between survivors and non-survivors among patients with device-related *E. faecium* BSI.

|  | **Survivors**  **N=101 (66.9%)** | **Non-survivors**  **N=50 (33.1%)** | **Overall**  **N=151 (100%)** | **p-value** |
| --- | --- | --- | --- | --- |
| **Demographic data** |  |  |  |  |
| Age (years) (median, IQR) | 68 (53 -77.5) | 72 (63.75-78) | 69 (56-78) | 0.018 |
| Male sex | 60 (59.4) | 32 (64.0) | 92 (60.9) | 0.601 |
| **Comorbidities** |  |  |  |  |
| Myocardial infarction | 14 (13.9) | 6 (12.0) | 20 (13.2) | 1.000 |
| Congestive heart failure | 16 (15.8) | 11 (22.0) | 27 (17.9) | 0.373 |
| Peripheral vascular disease | 7 (6.9) | 6 (12.0) | 13 (8.6) | 0.358 |
| CVA or TIA | 12 (11.9) | 5 (10.0) | 17 (11.3) | 1.000 |
| Dementia | 11 (10.9) | 4 (8.0) | 15 (9.9) | 0.774 |
| COPD | 13 (12.9) | 8 (16.0) | 21 (13.9) | 0.623 |
| Connective tissue disease | 5 (5.0) | 0 (0.0) | 5 (3.3) | 0.171 |
| Peptic ulcer disease | 7 (6.9) | 3 (6.0) | 10 (6.6) | 1.000 |
| Hemiplegia | 1 (1.0) | 2 (4.0) | 3 (2.0) | 0.255 |
| Moderate/severe CKD | 19 (18.8) | 13 (26.0) | 32 (21.2) | 0.397 |
| Leukemia | 9 (8.9) | 5 (10.0) | 14 (9.3) | 1.000 |
| Lymphoma | 4 (4.0) | 4 (8.0) | 8 (5.3) | 0.441 |
| Liver disease |  |  |  |  |
| Mild | 9 (8.9) | 4 (8.0) | 13 (8.6) | 1.000 |
| Moderate/severe | 11 (10.9) | 3 (6.0) | 14 (9.3) | 0.389 |
| Diabetes |  |  |  |  |
| Uncomplicated | 13 (12.9) | 9 (18.0) | 22 (14.6) | 0.464 |
| End-organ damage | 5 (5.0) | 1 (2.0) | 6 (4.0) | 0.664 |
| Solid tumor |  |  |  |  |
| Localized | 12 (11.9) | 4 (8.0) | 16 (10.6) | 0.581 |
| Metastatic | 8 (7.9) | 7 (14.0) | 15 (9.9) | 0.258 |
| Immunosuppression | 27 (26.7) | 14 (28.0) | 41 (27.2) | 1.000 |
| Neutropenia | 6 (5.9) | 10 (20.0) | 16 (10.6) | 0.012 |
| SOT | 11 (10.9) | 2 (4.0) | 13 (8.6) | 0.222 |
| HSCT | 4 (4.0) | 1 (2.0) | 5 (3.3) | 1.000 |
| Chronic steroid treatment | 10 (9.9) | 4 (8.0) | 14 (9.3) | 1.000 |
| COVID-19 during hospitalization | 22 (21.8) | 11 (22.0) | 33 (21.9) | 1.000 |
| CCI (median, IQR) | 5 (2.0-7.0) | 5 (4.0-8.25) | 5 (3.0-8.0) | 0.566 |
| **Characteristics of BSI** |  |  |  |  |
| Ward of BSI |  |  |  |  |
| Medical | 59 (58.4) | 25 (50.0) | 84 (55.6) | 0.053 |
| Surgical | 17 (16.8) | 3 (6.0) | 20 (13.2) |  |
| ICU | 21 (20.8) | 18 (36.0) | 39 (25.8) |  |
| Emergency department | 4 (4.0) | 4 (8.0) | 8 (5.3) |  |
| SOFA score (median, IQR) | 3 (1.0-5.0) | 6 (4.0-11.0) | 4 (2.0-6.0) | <0.001 |
| qSOFA score (median, IQR) | 0.00 (0.0-1.0) | 1.0 (0.0-2.0) | 0.0 (0.0-1.0) | <0.001 |
| PITT score (median, IQR) | 0.00 (0.0-1.0) | 2.0 (0.75- 4.0) | 1.0 (0.0- 2.0) | <0.001 |
| Septic shock | 4 (4.0) | 10 (20.0) | 14 (9.4) | 0.005 |
| Epidemiological classification |  |  |  |  |
| Healthcare-related | 10 (9.9) | 7 (14.3) | 17 (11.3) | 0.424 |
| Nosocomial | 91 (90.1) | 42 (85.7) | 133 (88.7) |  |
| **Microbiological characteristics** |  |  |  |  |
| VRE | 30 (29.7) | 14 (28.0) | 44 (29.1) | 1.000 |
| Monomicrobial | 62 (61.4) | 33 (66.0) | 95 (62.9) | 0.597 |
| Polymicrobial | 39 (38.6) | 17 (34.0) | 56 (37.1) |  |
| **Management of BSI** |  |  |  |  |
| FUBC | 50 (49.6) | 12 (24.0) | 62 (41.1) | 0.004 |
| Time from BSI to FUBC | 4 (2-5) | 4 (3-6) | 4 (2-5) | 0.211 |
| Time from BSI to negative FUBC | 4 (3-6) | 5 (3-6) | 4 (3-6) | 0.567 |
| Echocardiographic study | 60 (60.0) | 16 (32.0) | 76 (50.7) | 0.002 |
| Positive | 2 (3.3) | 1 (6.3) | 3 (3.9) | 0.513 |
| Ultrasound study | 32 (31.7) | 8 (16.3) | 40 (26.7) | 0.085 |
| Positive | 13 (40.6) | 4 (50.0) | 17 (42.5) | 0.702 |
| Device removal |  |  |  |  |
| Performed | 96 (95.0) | 31 (62.0) | 127 (84.1) | <0.001 |
| Not performed | 5 (5.0) | 19 (38.0) | 24 (15.9) |  |
| Time from BSI to source control (days) (median, IQR) | 3 (1.0- 5.75) | 2 (1.0-4.0) | 3 (1.0-5.0) | 0.541 |
| **Antibiotic treatment** |  |  |  |  |
| Empirical treatment | 73 (72.3) | 47 (94.0) | 120 (79.5) | 0.002 |
| Appropriate empirical therapy | 28 (38.4) | 18 (39.1) | 46 (38.7) | 1.000 |
| Targeted treatment | 71 (71.0) | 40 (80.0) | 111 (74.0) | 0.324 |
| Vancomycin | 12 (11.9) | 7 (14.0) | 19 (12.6) | 0.796 |
| CI after LD | 5 (41.7) | 1 (14.3) | 6 (31.6) | 0.420 |
| Intermittent infusion | 6 (50.0) | 6 (85.7) | 12 (63.2) |  |
| Extended infusion | 1 (8.3) | 0 (0.0) | 1 (5.3) |  |
| Teicoplanin | 25 (24.8) | 9 (18.0) | 34 (22.5) | 0.412 |
| Daptomycin | 5 (5.0) | 10 (20.0) | 15 (9.9) | 0.007 |
| <10 mg/Kg | 2 (40.0) | 7 (77.8) | 9 (64.3) | 0.266 |
| ≥10 mg/Kg | 3 (60.0) | 2 (22.2) | 5 (35.7) |  |
| Linezolid | 27 (26.7) | 14 (28.0) | 41 (27.2) | 1.000 |
| Monotherapy | 56 (55.4) | 30 (60.0) | 86 (57.0) | 0.606 |
| Combination therapy | 15 (14.9) | 10 (20.0) | 25 (16.6) | 0.487 |
| Beta-lactam | 13 (12.9) | 9 (18.0) | 22 (14.6) | 0.464 |
| Fosfomycin | 0 (0.0) | 1 (2.0) | 1 (0.7) | 0.331 |
| Appropriate targeted therapy | 69 (97.2) | 39 (97.5) | 108 (97.3) | 1.000 |
| Duration of appropriate therapy (days) (median, IQR) | 11.0 (6.0- 16.75) | 8.0 (5.5-11.5) | 10.0 (6.0-14.0) | 0.019 |
| Adverse event | 4 (6.3) | 1 (2.7) | 5 (5.0) | 0.650 |
| **Outcome** |  |  |  |  |
| Clinical cure (+7-day) | 87 (86.1) | 9 (18.0) | 96 (63.6) | <0.001 |
| SOFA score (+7-day) | 2.0 (1.0-4.0) | 7.0 (3.0- 9.25) | 3.0 (1.0-6.0) | <0.001 |
| Breakthrough BSI | 3 (3.0) | 1 (2.0) | 4 (2.6) | 1.000 |
| Recurrent BSI | 24 (23.8) | 6 (12.0) | 30 (19.9) | 0.128 |
| LOS (days) (median, IQR) | 47 (29-89.5) | 27.5 (17-59) | 40 (25-79) | 0.003 |
| Time from BSI onset to death (days) (median, IQR) |  | 11 (6.0-17.25) | 11 (6.0 -17.25) |  |

**Abbreviations:** CVA cerebrovascular accident, TIA transient ischemic attack, COPD chronic obstructive pulmonary disease, CKD chronic kidney disease, SOT solid organ transplant, HSCT hematologic stem cell transplant, CCI Charlson comorbidity score, BSI bloodstream infection, ICU intensive care unit, UTI urinary tract infection, FUBC follow-up blood cultures, CI continuous infusion, LD loading dose.
