# Supplementary figures and images for: Enterococcus faecium bacteraemia: a multicentre observational study focused on risk factors for clinical and microbiological outcomes
Source: J Antimicrob Chemother. 2025 Jun 19;80(8):2247–56. doi: 10.1093/jac/dkaf197 (PMC12313462; doi:10.1093/jac/dkaf197)

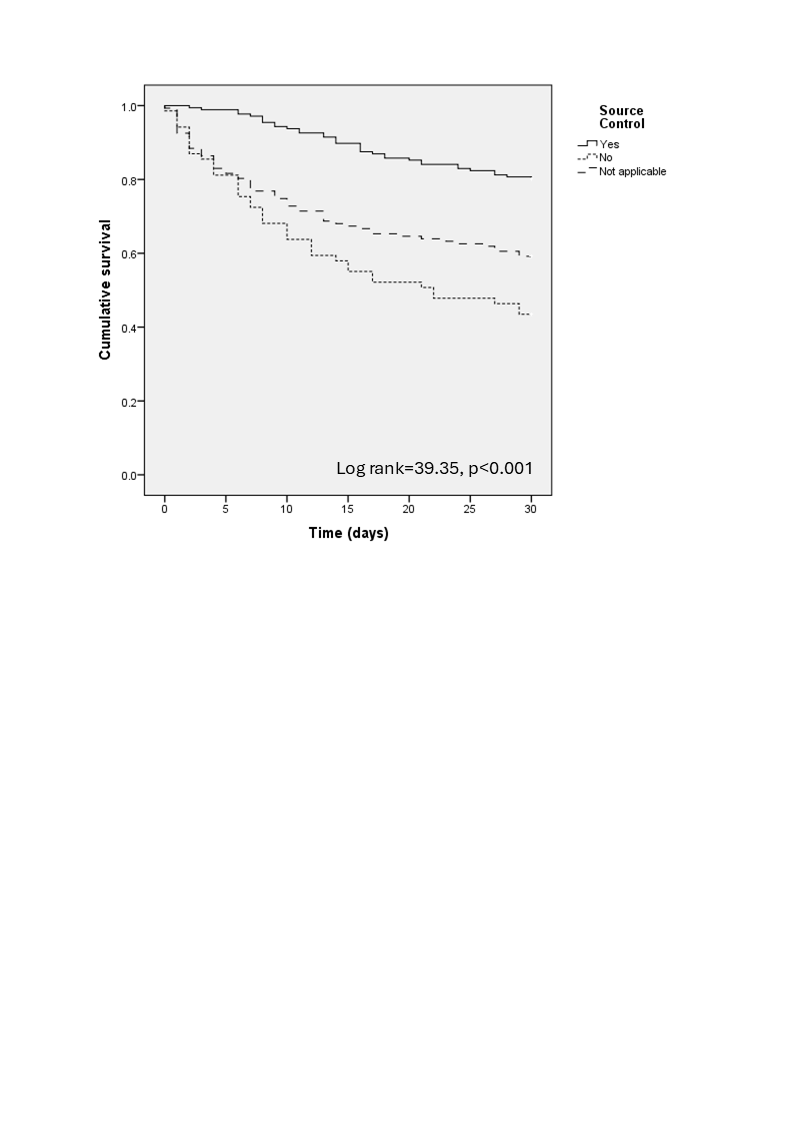

Supplement: dkaf197_Supplementary_Data [file dkaf197_supplementary_data.zip › Suppl. Figure 1.png]

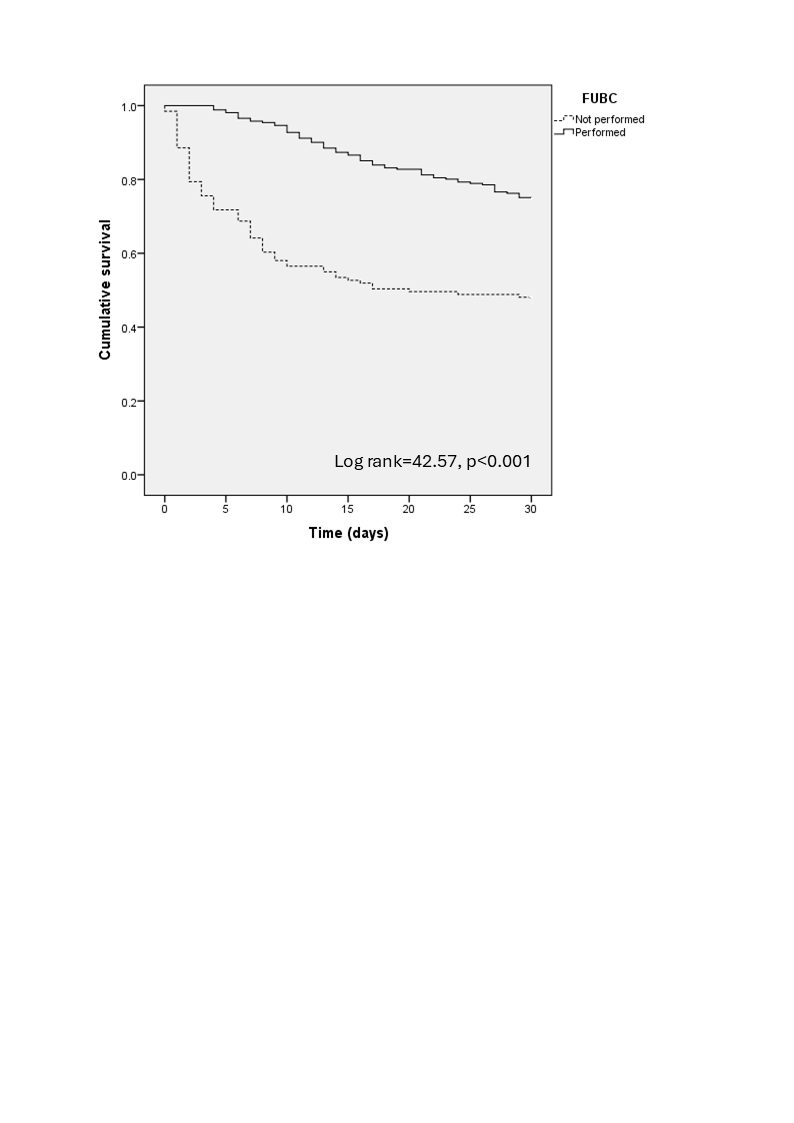

Supplement: dkaf197_Supplementary_Data [file dkaf197_supplementary_data.zip › Suppl. Figure 2.png]

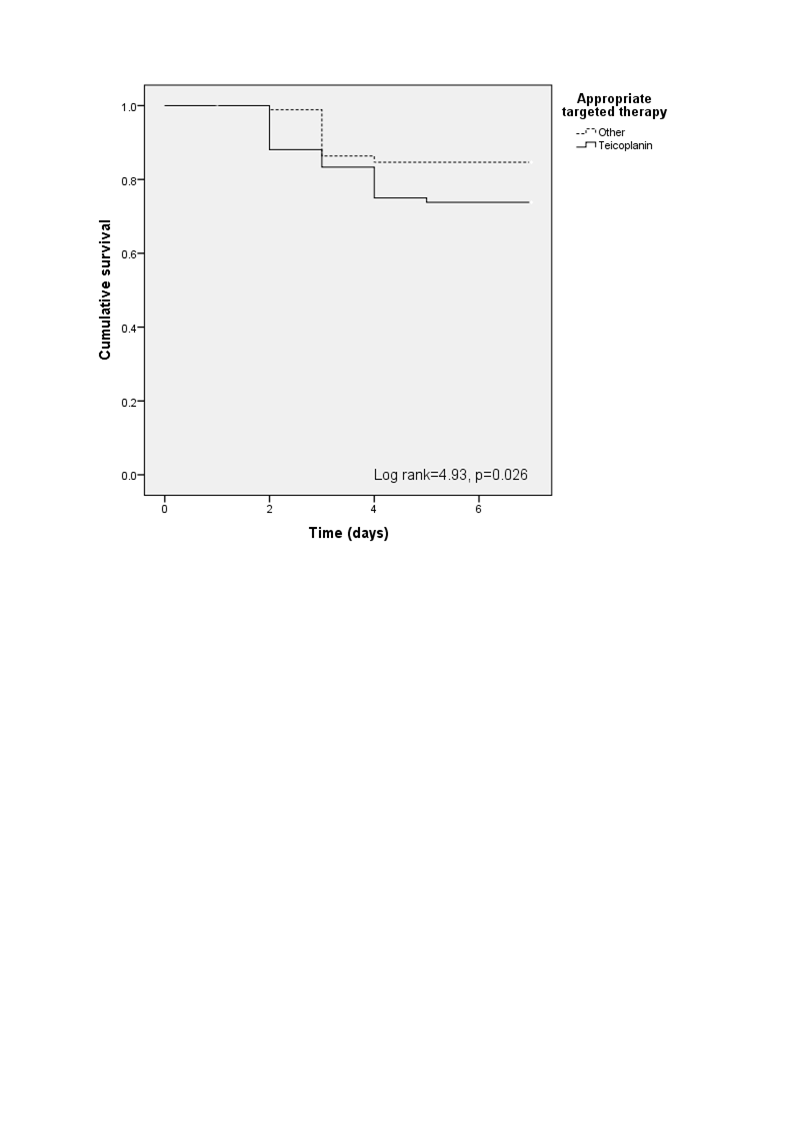

Supplement: dkaf197_Supplementary_Data [file dkaf197_supplementary_data.zip › Suppl. Figure 3.png]
